# Serum netrin and VCAM-1 as biomarker for Egyptian patients with type IΙ diabetes mellitus

**DOI:** 10.1016/j.bbrep.2021.101045

**Published:** 2021-06-11

**Authors:** Maher M. Fadel, Faten R. Abdel Ghaffar, Shimaa K. Zwain, Hany M. Ibrahim, Eman AE. badr

**Affiliations:** a Unit of Immunology and Physiology Department of Zoology, Faculty of Science, Menoufia University, Egypt; b Department of Internal Medicine, Diabetes and Endocrinology, Faculty of Medicine, Menoufia University, Egypt; c Department of Medical Biochemistry and Molecular Biology, Faculty of Medicine, Menoufia University, Egypt

**Keywords:** Diabetes, Netrin1, VCAM1, Complications

## Abstract

**Objective:**

This study aimed to evaluate the serum level of netrin and soluble vascular cell adhesion molecule 1 (VCAM-I) in patients with type IΙ diabetes mellitus (T2DM) and evaluate the association of their levels with the development of a diabetic complication.

**Patients and methods:**

This study was carried out on type II diabetic patients with and without complications and healthy individuals served as controls. All subjects were submitted to the estimation of serum lipid profile, serum creatinine, urinary albumin/creatinine ratio (ACR), fasting blood glucose (FBG), glycated hemoglobin (HbA1c), visceral adiposity index (VAI), atherogenic index of plasma (AIP), lipid accumulation product (LAP) and detection of serum level of netrin1 and VCAM1.

**Results:**

Diabetic patients with complications had significantly higher serum levels of creatinine, ACR, cholesterol, Triglyceride, low-density lipoprotein, netrin1, and VCAM1 than diabetic patients without complications. Likewise, the level of VAI and LAP as markers of excessive body fat were significantly higher in diabetic patients with complications than diabetic patients without complications. The netrin1 and VCAM1 were a significant discriminator of T2DM renal complications with a sensitivity of 96%, 90%, and specificity of 82.7%, 91.3% respectively.

**Conclusion:**

It can be concluded that serum netrin1 and VCAM1 correlated significantly with markers of excessive body fat, a renal complication in the patient with type 2 diabetes mellitus.

## 1 Introduction

Type 2 Diabetes mellitus (T2DM), a metabolic disease with global epidemiology that is associated with increased risk of cardiovascular disease, kidney disease, high mortality and morbidity, premature death**)** [1], T2DM is also associated with both macrovascular and microvascular complications. Chronic hyperglycemia has been identified as the primary factor that contributes to the development of diabetic vascular complications [3]. It is well-established that T2DM is associated with a proinflammatory immune status and is accompanied by an increase in the level of circulating inflammatory markers [4].

Studies suggest that low-grade inflammation, characterized by the production of cytokines, chemokines, and adipokines, is involved in the pathogenic processes that cause T2DM and its complications [5]. The release of inflammatory mediators such as cytokine can be mediated by hyperglycemia and oxidative stress. Chronic inflammation and oxidative stress are inextricably linked via complex interactions of both mutual amplification in the pathophysiology of diabetes [6].

The understanding of the inflammatory mechanisms involved in the development and progression of this disease will enable the identification of new potential targets and facilitate the design of innovative anti-inflammatory therapeutic strategies [7]. Five kinds of netrin have been identified for mammals (1, 3, 4, G1, and G2). All netrins are members of the superfamily of laminin. Netrin1 is a laminin-like protein with a molecular weight of 50–75 kD [8].

Netrin1 is of critical importance in the cardiovascular system. Recent studies have revealed the pro-angiogenic, anti-apoptotic, and anti-inflammatory properties of netrin-1 [9]. In addition, netrin1 was reported to be involved in leukocyte migration in peripheral organs, tissue regeneration, and the modulation of inflammation-based conditions [10]. Netrin1 related axonal functions have been linked to two classes of receptors: the deleted in colorectal cancer (DCC) family, including DCC and its orthologue Neogenin-1, and the Unc5s family, including Unc5A [11].

Vascular cell adhesion molecule1(VCAM1) is a molecule expressed on the surface of activated endothelium and a variety of other cell types including bone marrow fibroblasts, tissue macrophages, and dendritic cells. It can be upregulated by inflammatory mediators such as interleukin-1β (IL-1β), IL-4, CD44, tumor necrosis factor-α (TNF-α), and interferon- γ (IFN- γ). VCAM1 is a ligand for leukocytic integrins α4 β1 (VLA-4) in cells including eosinophils and α4 β7 integrins at the activated T-cells at the periphery [12]. Cross-sectional studies have shown an elevation in the levels of circulating VCAM1 in patients with diabetic nephropathy [13]. Nevertheless, there is still scarcity in the published literature that assesses the role of netrin1 and VCAM1 as biomarkers of diabetes and long-term complications. Therefore, we conducted the present study to evaluate the serum level of Netrin and soluble VCAM1 in patients with T2DM and evaluate the association of their levels with the development of diabetic complications.

## 2 Subjects and methods

A case-control study was carried out on 100 diabetic patients with T2DM_who were selected from the internal medicine department at Menoufia University hospital, Egypt and 80 normal healthy controls.

### 2.1 Study groups

The subjects were divided into 3 groups. Group 1, included 80 normal healthy participants, who age and sex matched to the patients groups. Group 2, included 50 diabetic patients without complications, diagnosed by clinical symptoms, fasting blood sugar (FBS) ≥126 mg/dl and/or post-prandial (PPBS) and/or random blood sugar (RBS) ≥200 mg/dl and/or glycated hemoglobin (HbA1c) level ≥ 6.5%,Group 3, included 50 diabetic patients with complications in the form of diabetic nephropathy diagnosed by the presence of microalbuminuria and/or reduced estimated glomerular filtration rate (eGFR) in the absence of signs or symptoms of other primary causes of kidney damage.

The diagnosis of DM and its chronic complications were established according to the criteria of the American Diabetes Association (ADA) criteria [2]. Patients with current renal or hepatic disease, malignancy, diabetes other than type 2 diabetes mellitus, any acute inflammation or infection, current significant cardiovascular disease were excluded from participation.

The protocol was approved by the Research Ethical Committee of faculty of Medicine, Menoufia University. Written informed consent was obtained from each participant.

All participants were subjected to full history taking, including sociodemographic characteristics, concomitant diseases, medications, macro-and microvascular complications of T2DM, and family history. Physical examination included anthropometric data, body composition, and blood pressure was performed. Blood pressure was measured using an aneroid sphygmomanometer.

### 2.2 Anthropometric measurements and indices

Estimation of body weight in kilograms and height in centimeters, then body mass index (BMI) was calculated as body weight in kilograms divided by squared height in meters. The waist circumference (WC), measured at the top of the iliac crests, and hip circumference (HC) measured at the largest part of the buttocks, were taken with light clothes using a non-stretchable tape; the values were approximated to the nearest 0.1 cm Calculated the waist-hip ratio (WHR) was calculated as the waist circumference to the hip circumference. Visceral adiposity index (VAI), calculated by using body mass index (BMI), WC, triglyceride (TG), and high-density lipoprotein-cholesterol (HDL-cholesterol) levels, is used successfully for detecting the distribution and function of visceral fat, insulin resistance (IR), and increased cardiometabolic risk.

Measuring systolic and diastolic blood pressure using a sphygmomanometer and stethoscope as the ‘gold standard for office blood pressure measurement, by an observer listening for the Korotkoff sounds. Hypertension was accomplished when systolic BP; ≥ 140 mm Hg and diastolic BP; ≥ 90 mm Hg [15,16].

VAI levels were calculated for women and men by the following formula: (WC/[36.58 + (1.89 × BMI)]) × [(TG (mmol/L)/0.81) × (1.52/HDL-c (mmol/L))] and (WC/[39.68 + (1.88 × BMI)]) × [(TG (mmol/L)/1.03) × (1.31/HDL-c (mmol/L))], respectively [17].

Lipid accumulation product (LAP) was estimated by the following equation; [(WC in cm − 65) × TG in mmol/L] in males and [(WC in cm − 58) × TG in mmol/L] in females [18].

The atherogenic index of plasma (AIP) is a good predictor of the risk of atherosclerosis and coronary heart disease AIP was calculated as log (TG/HDL–C) [19].

### 2.3 Sample collection & biochemical analysis

Eight ml of venous blood were withdrawn from every overnight (12 h) fasting subjects. The samples were divided into two fractions: 2.5 ml of blood were transferred into an EDTA tube as an anticoagulant for estimation of blood HA1c by quantitative colorimetric determination method [20]. Four ml of blood were transferred into a plain tube left to clot for 30 min, then centrifuged at 3000 rpm for 10 min, the serum obtained was kept frozen at - 20 °C for determination of serum creatinine by fixed-rate kinetic chemical method [21], using (Diamond diagnostics kit, Germany). eGFR (mL/min/1.73 m^2^) was calculated using the equation proposed by the Chronic Kidney Disease Epidemiology Collaboration study [22].

Measurement of lipid profile [23]: low-density lipoprotein cholesterol (LDLc), high-density lipoprotein cholesterol (HDLc), total cholesterol (TC), and triacylglycerol (TG) were determined using standard enzymatic colorimetric kits (Spinreact diagnostics kit, Spain).

One and a half ml of blood was transferred into a sodium fluoride tube for colorimetric determination of fasting blood glucose and 2 ml blood was collected after 2 h for colorimetric determination of postprandial blood glucose [24], using the glucose oxidase method (Spinreact diagnostics kit, Spain). Random urine samples were collected for determination of creatinine and microalbumin in urine to calculate the A/C ratio using DRG® Micro-Albuminuria ELISA kit, USA [25]. Measurement of serum Netrin1 [26] and VCAM1 [27] were done by ELISA technique using kits provided by MyBioSource Company, United States and Quantikine, R&D Systems, Inc. USA, respectively.

### 2.4 Statistical analysis

Data interpretation was performed by SPSS v25 (SPSS Inc., Chicago, IL, USA). Shapiro-Wilk test was used to verify the normality of distribution. Qualitative data were described using number and percent and were compared using the chi-square test or Fisher exact test. Quantitative parametric variables were presented as mean and standard deviation (SD). Comparisons were performed by ANOVA followed by Turkey post-test and Kruskal-Wallis test with Dunn's post-test. P ≤ 0.05 was taken as indicative of statistical significance. Spearman coefficient was used to correlate between two quantitative variables.

Receiver operating characteristic (ROC) curve generated by plotting sensitivity on the Y-axis versus specificity on the X-axis at different estimated values. The area under the ROC curve indicates test performance. For the test area, more than 50% reveals significant performance, and are about 100% reports the best performance. Positive Predictive Value (PPV) is the probability of the disease being present, among those with positive diagnostic test results. Negative Predictive Value (NPV) is the probability that the disease was absent, among those whose diagnostic test results were negative.

## 3 Results

Analysis of data obtained in this study revealed no significant difference between the three studied groups in terms of age, sex and height. There was a significant difference between the three studied groups in terms of weight (P > 0.001), body mass index (P > 0.001), waist circumference (P > 0.001), hip circumference (P > 0.001), waist/hip ratio (P > 0.001), systolic blood pressure (P > 0.02). Group II T2DM without complication and Group III T2DM with complication had a significant increase in terms of weight, body mass index, waist circumference, hip circumference, and waist/hip ratio compared to the control group. Group III T2DM with complication had also a significant increase in systolic blood pressure compared with Group I control (Table 1).

**Table 1:** Demographic and anthropometric data of controls and T2DM patients with or without complications

Measures	Group I Control (n = 80)	Group II T2DM without complication (n = 50)	Group III T2DM with complication (n = 50)
	No %	No %	No %
**Sex**Male	14(17.5%)	14(28%)	8(16%)
Female	66(82.5%)	36 (72%)	42(84%)
**Medications**			
**Insulin**	–	10(20%)	9(18%)
**Oral hypoglycemia drugs**	–	40(80%)	41(82%)
	**Mean** ± **SD**	**Mean** ± **SD**	**Mean** ± **SD**
**Age (years)**	52.22 ± 8.72	51.80 ± 6.84	48.92 ± 8.05
**Weight (kg)**	61.25 ± 4.60	100.13 ± 6.558#	101.38 ± 8.21#
**Height (cm)**	162.74 ± 4.87	164.43 ± 4.54	162.51 ± 3.03
**BMI (kg/m**^**2**^**)**	23.13 ± 1.53	37.09 ± 2.77#	38.48 ± 4.01#
**Waist circumference (cm)**	64.7 ± 4.38	106.86 ± 5.10*	108.98 ± 7.08*
**Hip circumference (cm)**	84.33 ± 4.53	117.20 ± 4.73*	118.38 ± 6.60*
**Waist/Hip ratio**	0.76 ± 0.027	0.91 ± 0.02*	0.92 ± 0.02*
**SBP (mmHg)**	114 ± 6.75	114.80 ± 7.35	117.80 ± 7.37*
**DBP (mmHg)**	75.8 ± 3.82	74.40 ± 5.77	74.60 ± 6.05
**Visceral adiposity index (VAI)**	1.32 ± 0.29	2.51 ± 0.44*	2.90 ± 0.68*$
**Atherogenic index of plasma**	−0.02 ± 0.06	0.17 ± 0.07#	0.21 ± 0.10#
**Lipid accumulation product (LAP)**	7.30 ± 7.62	70.96 ± 13.65#	84.43 ± 17.88#$

SD: standard deviation, SBP: systolic blood pressure, DBP: diastolic blood pressure, BMI: body mass index.

Data are expressed as mean ± standard deviation or number of patients (% among study population).

(*) indicate significant *P < 0.05* difference compared with group I control using ANOVA.

(#) indicate significant *P < 0.05* difference compared with group I control using the Kruskal-Wallis test.

($) indicate significant *P < 0.05* difference compared with group II T2DM without complication.

On the levels of indices, Group II T2DM without complication and Group III T2DM with complication had a significant increase in terms of visceral adiposity index (VAI), Atherogenic index of plasma, and Lipid accumulation product (LAP) compared with the control group. Group III T2DM with complication had a significant increase in terms of visceral adiposity index (VAI), and Lipid accumulation product (LAP) compared with Group II T2DM without complication, as shown in table (1).

Regarding diabetic profile characteristics, there was a significant difference between the three studied groups in terms of fasting blood sugar (FBS), 2 h postprandial blood sugar (2HPPBS), and glycated hemoglobin percent (HbA1c%) (Table 2). Group II T2DM without complication and Group III T2DM with complication had a significant increase in FBS, 2HPPBS, and HbA1c percent compared with the control group. Group III T2DM with complication had a significant decrease in 2HPPBS compared with Group II T2DM without complication, as shown in Table (2).

**Table 2:** Fasting blood sugar, (mg/dl) 2HPPBS (mg/dl) and HbA1c (%) of controls and T2DM patients with or without complications

	Group I Control (n = 80)	Group II T2DM without complication (n = 50)	Group III T2DM with complication (n = 50)
	Mean ± SD	Mean ± SD	Mean ± SD
**FBS (mg/dl)**	91.5 ± 5.17	177.64 ± 28.1*	180.78 ± 15.32*
**2HPPBS (mg/dl)**	124.22 ± 8.35	291.02 ± 43.61*	260.88 ± 35.65*$
**HbA1c (%)**	4.82 ± 0.31	8.53 ± 0.80*	8.77 ± 0.60*

Data are expressed as mean ± standard deviation.

FBS: fasting blood sugar, 2HPPBS: 2 h postprandial blood sugar, HbA1c: glycated hemoglobin.

(*) indicate significant *P < 0.05* difference compared with group I control using ANOVA.

($) indicate significant *P < 0.05* difference compared with group II T2DM without complication**.**

On the levels of renal function and lipid profile, Group II T2DM without complication had a significant increase in terms of cholesterol and LDL, however, it had a significant decrease in HDL compared with the control group. Group III T2DM with complication had a higher significant increase in terms of creatinine, ACR, cholesterol, TG, and LDL, however, it had a significant decrease in HDL compared with the control group. Moreover, Group III T2DM with complication had a significant increase in terms of creatinine and ACR compared with Group II T2DM without complication, as shown in Table (3).

**Table 3:** Renal functions and lipid profile of controls and T2DM patients with or without complications.

	Group I Control (n = 80)	Group II T2DM without complication (n = 50)	Group III T2DM with complication (n = 50)
	Mean ± SD	Mean ± SD	Mean ± SD
**Creatinine (mg/dl)**	0.71 ± 0.10	0.88 ± 0.12	1.30 ± 0.41#$
**ACR (ug/mg)**	16.7 ± 4.52	18.02 ± 3.25	338.66 ± 53.61#$
**Cholesterol (mg/dl)**	141.27 ± 19.45	192.09 ± 19.37#	191.34 ± 31.55#
**TG (mg/dl)**	127.5 ± 12.17	134.12 ± 19.51	150.62 ± 26.77#
**LDLc (mg/dl)**	57.29 ± 20.014	126.76 ± 23.0#	124.58 ± 34.0#
**HDLc (mg/dl)**	58.8 ± 5.69	39.46 ± 3.08*	40.33 ± 2.91*

Data are expressed as mean ± standard deviation or number of patients (% among study population).

(*) indicate significant *P < 0.05* difference compared with group I control using ANOVA.

(#) indicate significant *P < 0.05* difference compared with group I control using the Kruskal-Wallis test.

($) indicate significant *P < 0.05* difference compared with group II T2DM without complication.

**Fig. 1:**
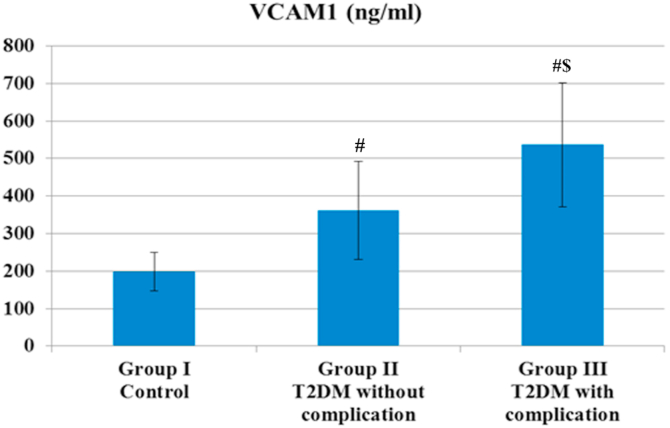
**Vascular cell adhesion molecule 1 in the three studied groups.** (#) indicate significant *P < 0.05* difference compared with group I controlusing Kruskal-Wallis test. ($) indicate significant *P < 0.05* difference compared with group II T2DM without complication.

**Fig. 2:**
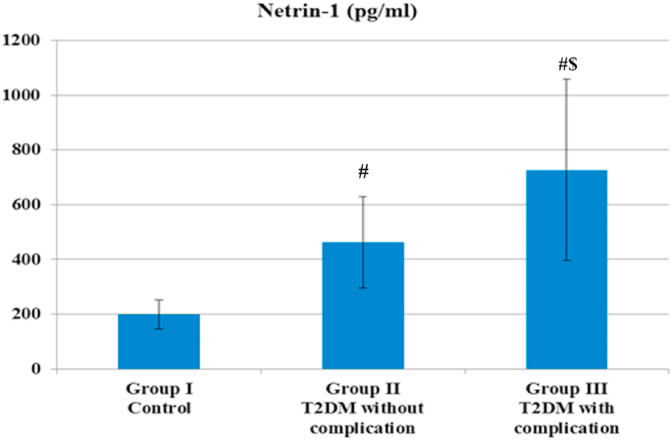
**Netrin 1 in the three studied groups.** (#) indicate significant *P < 0.05* difference compared with group I controlusing Kruskal-Wallis test. ($) indicate significant *P < 0.05* difference compared with group II T2DM without complication.

Finally, Group II T2DM without complication and Group III T2DM with complication had a significant increase in terms of VCAM1 and netrin1 compared with the control group. Group III T2DM with complication had a significant increase in terms of VCAM1 and netrin1 compared with Group II T2DM without complication, as illustrated in Fig. 1, Fig. 2.

To detect the correlation among VCAM1 or netrin and the different examined parameters the spearman test was conducted. The VCAM1 level was significantly and positively correlated with body mass index and HDL in the control group. While its level significantly and negatively correlated with GFR and significantly and positively correlated with ACR, respectively, in-group II T2DM without complication. The VCAM1 level significantly and negatively correlated with waist circumference and HDL, and significantly and positively correlated with HbA1C, TG, LDL, and atherogenic index of plasma in-group III T2DM with complication, as shown in Table (4).

**Table 4:** Correlation between VCAM1 with different parameters in each examined group

	Group I Control (n = 80)		Group II T2DM without complication (n = 50)		Group III T2DM with complication (n = 50)	
	r_s_	P	r_s_	p	r_s_	p
**BMI (kg/m2)**	0.287	0.004*	0.256	0.073	−0.149	0.303
**Waist circumference (cm)**	0.189	0.091	0.070	0.632	−0.285	0.045*
**HbA1c (%)**	0.070	0.475	0.184	0.200	0.476	<0.001*
**GFR (ml/min/1.73m**^**2**^**)**	0.165	0.105	−0.309	0.029*	−0.049	0.734
**ACR (ug/mg)**	−0.059	0.573	0.431	0.002*	0.050	0.731
**TG (mg/dl)**	0.025	0.840	−0.072	0.621	0.358	0.011*
**HDL (mg/dl)**	0.206	0.040*	−0.184	0.201	−0.416	0.003*
**LDL (mg/dl)**	−0.154	0.133	−0.202	0.160	0.279	0.049*
**Atherogenic index of plasma**	−0.125	0.230	0.037	0.797	0.344	0.015*

**r**_**s**_**: Spearman coefficient** *: Statistically significant at *P ≤ 0.05*.

The Netrin1 level significantly and negatively correlated with weight, body mass index, waist circumference, hip circumference, and HDL, however Netrin1 level significantly and positively correlated with systolic blood pressure, diastolic blood pressure, HbA1c, LDLc, TG, visceral adiposity index, and atherogenic index of plasma in-group III T2DM with complication, as shown in Table (5).

**Table 5:** Correlation between Netrin1 with different parameters in all studied group

	Group I Control (n = 80)		Group II T2DM without complication (n = 50)		Group III T2DM with complication (n = 50)	
	r_s_	p	r_s_	p	r_s_	p
**Weight (kg)**	0.080	0.410	0.013	0.927	−0.384	0.006*
**BMI (kg/m2)**	0.155	0.119	−0.020	0.892	−0.319	0.024*
**Waist circumference (cm)**	−0.016	0.909	0.005	0.972	−0.486	<0.001*
**Hip circumference (cm)**	0.0145	0.904	0.078	0.590	−0.468	0.001*
**SBP (mmHy)**	−0.026	0.775	0.081	0.576	0.309	0.029*
**DBP (mmHy)**	0.033	0.734	−0.085	0.558	0.324	0.022*
**HbA1c (%)**	0.025	0.785	0.241	0.091	0.489	<0.001*
**HDL (mg/dl)**	−0.110	0.250	−0.262	0.066	−0.518	<0.001*
**LDL (mg/dl)**	−0.009	0.943	0.106	0.465	0.300	0.034*
**TG (mg/dl)**	−0.103	0.296	−0.121	0.404	0.408	0.003*
**Visceral adiposity index (VAI)**	0.095	0.326	−0.117	0.418	0.399	0.004*
**Atherogenic index of plasma**	−0.035	0.709	−0.038	0.794	0.415	0.003*

**r**_**s**_**: Spearman coefficient** *: Statistically significant at *P ≤ 0.05*.

**Fig. 3:**
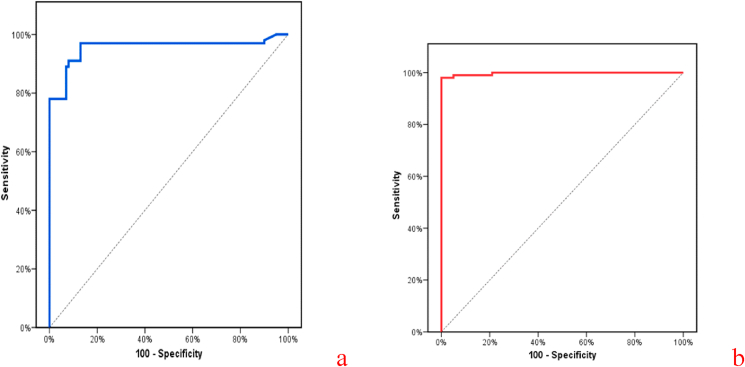
ROC curve for a-VCAM1 (ng/ml) b- Netrin1(pg/ml) to diffrentiate patients from control.

To differentiate between T2DM patients and the control group, The ROC curve showed that the VCAM1 was a significant discriminator of T2DM with a combined sensitivity and specificity of 97% and 87% with a cutoff point of more than 240 (ng/ml). In addition, Netrin1 was a significant discriminator of T2DM with a combined sensitivity and specificity of 98% and 95% with a cutoff point of more than 292 (pg/ml), as shown in Table (6) and illustrated in figure (3) a & b.

**Table 6:** Agreement of sensitivity and/or specificity for VCAM1 (ng/ml) and Netrin1 (pg/ml)

	AUC	P	95% C.I		Cut off	Sensitivity	Specificity	PPV	NPV
			LL	UL					
To differentiate between patients and control									
**VCAM1 (ng/ml)**	0.95	<0.001^a^	0.92	0.98	>240	97.0	87.0	88.2	96.7
**Netrin1 (pg/ml)**	0.99	<0.001^a^	0.99	1.00	>292.01	98.0	95.0	95.1	97.9
**To differentiate between patients with and without complication**									
**VCAM1 (ng/ml)**	0.93	<0.001^a^	0.90	0.96	>390	90.0	91.33	77.6	96.5
**Netrin1 (pg/ml)**	0.93	<0.001^a^	0.90	0.96	>393.6	96.0	82.67	64.9	98.4

AUC: Area Under a Curve, P-value: Probability value, CI: Confidence Intervals, NPV: Negative predictive value, PPV: Positive predictive value.

a Statistically significant at P ≤ 0.05, Cut off was choose according to the Youden index.

**Fig. 4:**
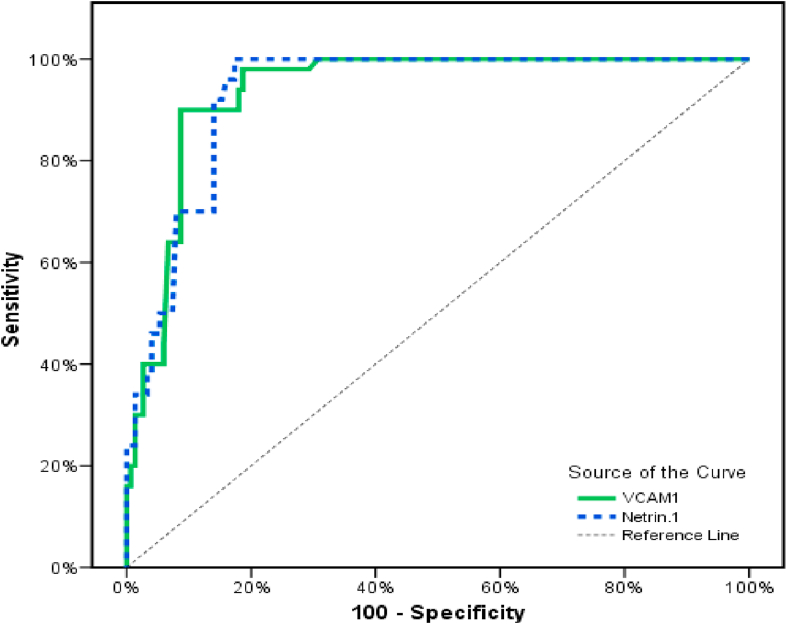
ROC curve for VCAM1 (ng/ml) and Netrin1 (pg/ml)to diffrentiate patients with complication.

To differentiate between T2DM with and without complication, The ROC curve showed that the VCAM1 was a significant discriminator of T2DM with complication, with a combined sensitivity and specificity of 90% and 91% with a cutoff point of more than 390 (ng/ml). In addition, Netrin1 was a significant discriminator of T2DM with complication, with a combined sensitivity and specificity of 96% and 82% with a cutoff point of more than 393 (pg/ml), as shown in Table (6) and illustrated in figure (4).

To detect the risk factors for renal complication, Univariate and multivariate logistic regression analysis showed that BMI, triglyceride, VAI, Atherogenic index of plasma and LAP act as independent risk factors while serum netrin1 and VCAM1 levels are dependant risk factors, as shown in Table (7).

**Table 7:** Univariate and multivariate logistic regression analysis

	Univariate		aMultivariate	
	p	OR (95%C.I)	p	OR (95%C.I)
**Age (years)**	**0.059**	0.949 (0.899–1.002)	**0.083**	0.921 (0.839–1.011)
**BMI (kg/m**^**2**^**)**	**0.049**^a^	1.127 (1.000–1.270)	**0.873**	0.953 (0.528–1.719)
**Cholesterol (mg/dl)**	**0.885**	0.999 (0.984–1.014)	**0.372**	0.959 (0.876–1.051)
**LDL (mg/dl)**	**0.705**	0.997 (0.984–1.011)	**0.203**	1.052 (0.973–1.139)
**TG (mg/dl)**	**0.002**^a^	1.033 (1.012–1.054)	**0.388**	0.870 (0.633–1.194)
**HDL (mg/dl)**	**0.150**	1.105 (0.965–1.265)	**0.33**	3.630(1.108–11.892)
**Visceral adiposity index (VAI)**	**0.003**^a^	3.882 (1.562–9.648)	**0.144**	6.599(0.524–83.168)
**Atherogenic index of plasma**	**0.036**^a^	153.5(1.376–17124.4)	**0.248**	3.026 (0–31.071)
**Lipid accumulation product (LAP)**	**<0.001**^a^	1.056 (1.025–1.087)	**0.959**	0.995 (0.828–1.197)
**Netrin1a (/pg/ml)**	**<0.001**^a^	1.570 (1.245–1.979)	**<0.001**^a^	3.083 (1.702–5.583)
**VCAM1 (/ng/ml)**	**<0.001**^a^	2.390 (1.606–3.556)	**<0.001**^a^	3.035 (1.638–5.622)

OR: Odd's ratio C.I: Confidence interval LL: Lower limit UL: Upper Limit.

a Statistically significant at p ≤ 0.05.

## 4 Discussion

Type 2 diabetes mellitus (T2DM) has been recognized as one of the leading causes of death and disabilities worldwide with an increasingly alarming rate. Uncontrolled or untreated T2DM leads to serious complications, including diabetic retinopathy, neuropathy, and nephropathy [28]. Obesity is the major reason behind insulin resistance which is mainly responsible for T2DM [29]**.** Early diagnosis of T2DM can reduce long-term complications, such as diabetic retinopathy, kidney failure, cardiovascular disease, and limb amputation. However, currently available screening tests such as the fasting plasma glucose (FPG) test, HbA1c test, and the oral glucose tolerance test (OGTT) exhibited low diagnostic accuracy for early detection of T2DM. Therefore, there is an unmet need for screening methods that can improve the early screening of T2DM such as genetic polymorphisms [30].

Netrin has recently received more attention as a biomarker of diabetes and a broader range of long-term complications. Therefore, netrin may be a potential biomarker [31]*.* On the other hand, VCAM1 is found on the walls of vessels, and it mediates the rolling and transendothelial migration of inflammatory cells into the intima by providing an anchor into the circulation. The endothelial activation may also play a role in diabetes. Thus, these processes not only affect the vascular endothelium as such but also contribute to the development of microvascular complications [32].

On the other hand, dyslipidemia is a common feature of diabetes. There is an association between atherosclerotic cardiovascular disease and serum cholesterol and triglyceride levels in both type 1 and type 2 diabetes. Moreover, overweight and obesity can lead to more deterioration of lipid profiles in T2DM [33]**.** Our analysis showed that there were statistically significant differences between cases and controls in terms of cholesterol, triglyceride, LDL, and HDL. Patients had significantly higher cholesterol levels, triglycerides, and LDL than the control group.

In agreement with our findings, [46] compared the lipid profile between diabetic, obese, patients with the non-diabetic population and found that obese T2DM patients showed a statistically significant increase in the levels of serum total cholesterol, serum triglycerides, and serum LDL-cholesterol. Similar results were obtained by Das and Banik [34].

In the past, abdominal obesity was detected by dual-energy X-ray absorptiometry (DEXA), computed tomography (CT), magnetic resonance imaging (MRI), and dual bioelectrical impedance analysis (BIA), which were unsuitable for routine clinical practices on account of the radiation exposure, time requirements, and high costs. Thus, nowadays many indices to estimate central or abdominal obesity has been established, including VAI and LAP [18].

To sum up our investigations of the association between T2DM and metabolic abnormalities, we compared the VAI between diabetes and the control group. We found that diabetic patients had statistically significantly higher levels of VAI, AIP, and LAP than the control group. In line with our findings, Nusrianto and his colleagues [35] performed a systematic review to inquire whether VAI can be used as a predictor of T2DM in the Asian population with different body composition compared to Caucasians. This study found that VAI can be used as a predictor of T2DM in the Asian population with better prediction values compared to the Caucasian population.

Likewise, Hameed and Abdulqahar [36], demonstrated that diabetic patients had statistically significantly higher levels of VAI than the control group. Another study also detected that diabetic patients had statistically significantly higher levels of AIP than the control group [19].

Renal impairment is one of the most feared complications in the setting of diabetes. Diabetic nephropathy is supposed to develop in nearly one-third of T2DM patients, which, in return, progresses to chronic kidney disease (CKD) then to chronic renal failure [14]. Several reports demonstrated that microalbuminuria, a well-established marker of renal impairment, is an early finding in obese patients with diabetic nephropathy; the degree of albuminuria was found to be significantly correlated with body weight in obese diabetic patients [37]. Moreover, microalbuminuria was found to be an early predictor of end-stage renal disease (ESRD) and cardiovascular diseases in these types of patients [38].

In our report, we found that diabetic patients had a significantly higher level of serum creatinine, albumin-creatinine ratio, and a lower level of glomerular filtration rate. In agreement with our findings, in ninety subjects with the age group 45–75 years of either gender, renal profile when compared, urea, creatinine, and potassium were significantly higher in T2DM with CKD as compared to controls and T2DM without CKD [39].

VCAM1 represents inducible endothelial cell adhesion molecules, where VCAM1 belongs to the Ig-superfamily. This molecule is expressed on endothelium in atherosclerotic plaques and has been shown to play an important role in the development of the atherosclerotic lesion [40]. In the present study, we found that diabetic patients had a significantly higher level of serum VCAM1 than the control group. The VCAM1 was a significant discriminator of T2DM with a sensitivity of 97% and specificity of 87%.

In concordance with our findings, Song and his colleagues [41] conducted a prospective nested case-control study to examine the associations between plasma levels of VCAM1 and diabetes risk among 82,069 initially healthy women aged 50–79 years from the Women's Health Initiative Observational Study. During a median follow-up of 5.9 years, 1584 incident diabetes case subjects were matched with 2198 control subjects by age, ethnicity, clinical center, time of blood draw, and follow-up time. Baseline median levels of the VCAM1 were each significantly higher among case subjects than among control subjects.

In the present study, we found that diabetic patients had a significantly higher level of serum netrin1 than the control group. The netrin1 was a significant discriminator of T2DM with a sensitivity of 98% and specificity of 95%. A recent clinical study by [44] claimed that netrin1 may be a new biomarker for early detection of impaired fasting glucose (IFG) or T2DM. Briefly, they found a significant increment of serum netrin1 levels in subjects with IFG or T2DM compared to the control group; serum netrin1 levels had a significant positive correlation with fasting glucose, HbA1c, HOMA-IR, AST, and ALT. Also, a statistically inverse correlation was found between netrin1 and HDL cholesterol and eGFR levels. On top of that, serum netrin1 was independently associated with the presence of IFG or T2DM [10].

On the contrary, [45] conducted a clinical study on 56 human subjects, where 30 subjects who had new-onset type 2 diabetes were allocated to the treatment group while the remaining were assigned to the control group to assess the extent of netrin1 in diabetic patients. They found that the level of Netrin-1 in diabetic patients was meaningfully reduced than that of healthy controls. Additionally, the extent of Netrin-1 was found to be inversely related to the homeostasis model evaluation of insulin resistance and plasma glucose (fasting and post-meal), fasting insulin, triglyceride, and hemoglobin A1c levels.

The exact causes of such heterogeneity in the published literature, regarding the association between netrin1 with T2DM, are unclear. However, this difference can be attributed to many methodological differences. Different populations, whether healthy subjects or T2DM patients, were included in the above-mentioned studies. Moreover, the types of assessment were substantially different among these published studies. Another explanation might be rendered to the differences in sample size.

Notably, our analysis demonstrated that serum netrin1 correlated significantly with markers of excessive body fat, such as VAI, atherogenic index of plasma, and LAP. To our knowledge, no clinical studies have assessed the correlation between serum Netrin and VAI yet. Our findings can be explained by the recent findings that the neuroimmune guidance cue netrin-1 contributes to maladaptive macrophage immune responses in obesity and atherosclerosis by fostering macrophage persistence in tissues. Macrophages accumulate prominently in the visceral adipose tissue (VAT) of obese humans and high-fat diet (HFD) fed mice, and this is linked to insulin resistance and type II diabetes [42].

The receptor protein tyrosine kinase family of ephrin (Eph) receptors and their ligands ephrins were implicated in monocyte activation and migration into inflamed tissue. It was demonstrated that ephrin-A1-induced EphA4 forward signaling in endothelial cells increases monocyte adhesion and ephrinA1-dependent activation of the EphA2 receptor influences the expression of VCAM1. Monocytes were activated by interaction with EphrinB2. Through EphB4 forward signaling and ephrin B2 reverse signaling, Ephrin B2–Eph B interaction was shown to influence monocyte adhesion and transendothelial migration. Netrin-1 is crucial in retaining monocytes and macrophages, preventing them from leaving atherosclerotic lesions [43].

Concerning the association between studied markers and the complications of T2DM, we found that diabetic patients with complications had a significantly higher level of serum netrin1 than the control group without complications. The Netrin was a significant discriminator of T2DM renal complications with a sensitivity of 96% and specificity of 82.7%. Likewise, diabetic patients with complications had a significantly higher level of VCAM1 than the control group without complications. VCAM1 was a significant discriminator of T2DM renal complications with a sensitivity of 90% and specificity of 91.3%.

Netrin1, the axon-guidance molecule has recently become an investigational protein in modulating inflammation, apoptosis, and many other pathological alterations in renal tubular epithelial cells. For instance, Netrin1 anti-inflammatory actions were mediated through diabetes-induced cyclooxygenase 2 (COX2) expression and prostaglandin E2 (PGE2) production. This suppressive effect of COX-2 was expedited through the inhibition of nuclear factor kappa-B (NF*κ*B). These inflammatory suppressant actions of Netrin-1 were proposed to modulate not only diabetic nephropathy but also the progression of various microvascular diabetic complications [31].

Plasma netrin-1 level may be associated with albuminuria and estimated glomerular filtration rate, independently of age and sex [8].

## 5 Conclusion

The VCAM1 and Netrin1 protein levels were significantly correlated with the presence of complications in T2DM. They achieved fair diagnostic accuracies for the prediction of diabetes and its complications. Augmented induction of endothelial VCAM1 expression by circulating factor(s) may play a role in the development of atherosclerosis in diabetes. On the other hand, Netrin1 is a potential biomarker and has a therapeutic implication in diabetes and diverse sets of microvascular and macrovascular complications of diabetes.

## Study's limitations

We acknowledge that the present study has some limitations. This was a cross-sectional study with inherent limitations of possible misclassification and ascertainment bias. Besides, the study was a single-center experience and therefore the results cannot be generalized to the general population.

## Author's contribution

HMI, FRA, and EAEB design study, EAEB and MMF do the lab investigation, HMI analyzes the results, SKZ collects the sample, and follows the patients. All authors write and revise the manuscript and approve the final manuscript for submission.

## Declaration of competing interest

The authors declare that they have no conflict of interest.
